# SGTA associates with intracellular aggregates in neurodegenerative diseases

**DOI:** 10.1186/s13041-021-00770-1

**Published:** 2021-03-23

**Authors:** Shun Kubota, Hiroshi Doi, Shigeru Koyano, Kenichi Tanaka, Hiroyasu Komiya, Atsuko Katsumoto, Shingo Ikeda, Shunta Hashiguchi, Haruko Nakamura, Ryoko Fukai, Keita Takahashi, Misako Kunii, Mikiko Tada, Hideyuki Takeuchi, Fumiaki Tanaka

**Affiliations:** 1 Department of Neurology and Stroke Medicine, Yokohama City University Graduate School of Medicine, 3-9 Fukuura, Kanazawa-ku, Yokohama, 236-0004 Japan; 2 Department of Neurology, Yokohama Minami Kyosai Hospital, 1-21-1 Mutuurahigashi, Kanazawa-ku, Yokohama, 236-0037 Japan

**Keywords:** SGTA, Polyglutamine disease, Neurodegeneration, Intranuclear inclusion bodies, Multiple system atrophy

## Abstract

Intracellular aggregates are a common pathological hallmark of neurodegenerative diseases such as polyglutamine (polyQ) diseases, amyotrophic lateral sclerosis (ALS), Parkinson’s disease (PD), and multiple system atrophy (MSA). Aggregates are mainly formed by aberrant disease-specific proteins and are accompanied by accumulation of other aggregate-interacting proteins. Although aggregate-interacting proteins have been considered to modulate the formation of aggregates and to be involved in molecular mechanisms of disease progression, the components of aggregate-interacting proteins remain unknown. In this study, we showed that small glutamine-rich tetratricopeptide repeat-containing protein alfa (SGTA) is an aggregate-interacting protein in neurodegenerative diseases. Immunohistochemistry showed that SGTA interacted with intracellular aggregates in Huntington disease (HD) cell models and neurons of HD model mice. We also revealed that SGTA colocalized with intracellular aggregates in postmortem brains of patients with polyQ diseases including spinocerebellar ataxia (SCA)1, SCA2, SCA3, and dentatorubral–pallidoluysian atrophy. In addition, SGTA colocalized with glial cytoplasmic inclusions in the brains of MSA patients, whereas no accumulation of SGTA was observed in neurons of PD and ALS patients. In vitro study showed that SGTA bound to polyQ aggregates through its C-terminal domain and SGTA overexpression reduced intracellular aggregates. These results suggest that SGTA may play a role in the formation of aggregates and may act as potential modifier of molecular pathological mechanisms of polyQ diseases and MSA.

**Supplementary Information:**

The online version contains supplementary material available at 10.1186/s13041-021-00770-1.

## Introduction

Formation of intracellular protein aggregates, which are microscopically observed as inclusion bodies, is a common pathological feature of neurodegenerative diseases including polyglutamine (polyQ) diseases, amyotrophic lateral sclerosis (ALS), Parkinson’s disease (PD), and multiple system atrophy (MSA) [1, 2]. Although the main components of protein aggregates differ in each disease, the formation process of protein aggregates is thought to share common molecular and pathological pathways.

In polyQ diseases, such as Huntington disease (HD) and several spinocerebellar ataxias (SCAs), aberrant proteins are produced by each causative gene harboring abnormally expanded polyQ tracts. These aberrant proteins change their conformation, repeat self-polymerization, and finally form insoluble aggregates [3]. PolyQ aggregates are also composed of numerous aggregate-interacting proteins. Some of these aggregate-interacting proteins are considered to be related to the process of aggregate formation and are widely associated with pathophysiology of various neurodegenerative diseases. For instance, Hdj1, an Hsp40 family chaperone protein, has been reported to interact with polyQ aggregates in HD model cells and animals, and suppress aggregate formation and reduce cellular toxicity in vitro [4]. It has also been reported that sequestration of Hsp70 and Sis1 into polyQ aggregates disturbs ubiquitin–proteasome-mediated protein degradation and impedes intracellular protein homeostasis [5]. Ubiquilins and FET (also called TET) family proteins, which are components of polyQ aggregates [6, 7], are also related to ALS. Thus, the study of aggregate-interacting proteins could help to uncover molecular pathological mechanisms common to several neurodegenerative diseases.

Previously, we identified small glutamine-rich tetratricopeptide repeat (TPR)-containing protein alpha (SGTA) as a component of polyQ aggregates in HD model cells (unpublished data). SGTA is a cochaperone that interacts with various proteins including heat shock proteins [8]. SGTA is particularly important for intracellular protein homeostasis and associates with mislocalization of membrane proteins with hydrophobic residues, such as substrates for endoplasmic reticulum-associated degradation (ERAD) [9, 10]. Although Sgt2, a yeast ortholog of SGTA, has been shown to interact with polyQ aggregates in yeast [11, 12], there are currently no studies that have demonstrated the association of SGTA with intracellular aggregates of human neurodegenerative diseases. The purpose of this study was to evaluate the localization of SGTA in cell and animal models, in postmortem brains of patients with polyQ diseases, PD, and MSA, and in the spinal cord of ALS patients.

## Materials and methods

### Antibodies

We used the following antibodies: polyclonal anti-SGTA antibody (TA308276, 1:200 for immunocytochemistry and immunohistochemistry of human tissue; Origene Technologies, Rockville, MD), monoclonal anti-SGTA antibody (sc-374031, 1:250 for western blotting, 1:100 for immunohistochemistry of mouse tissue; Santa Cruz Biotechnology, Santa Cruz, CA), monoclonal anti-ubiquitin antibody (MAB1510, 1:250 for immunocytochemistry; Chemicon, Temecula, CA), polyclonal anti-Lamin B (M-20) antibody (sc-6217, 1:250 for western blotting; Santa Cruz Biotechnology), monoclonal anti-expanded polyQ antibody (1C2, MAB1574, 1:5000 for immunohistochemistry; Chemicon), monoclonal anti-BAG6 antibody (sc-365928, 1:250; Santa Cruz Biotechnology), monoclonal anti-phosphorylated-α-synuclein antibody (pSyn#64, 1:400; Wako Chemicals, Tokyo, Japan), monoclonal anti-phosphorylated TDP-43 antibody (TIP-PTD-M01, 1:1000; Cosmo Bio Co, Tokyo, Japan), polyclonal anti-V5 antibody (A190-120A, 1:500; Wako Chemicals, Montgomery, TX), polyclonal anti-GFP antibody (598, 1:500; Medical and Biological Laboratories, Nagoya, Japan), HRP-conjugated anti-mouse IgG and anti-rabbit IgG antibodies (NA931 and NA934, 1:5000; GE Healthcare, Buckinghamshire, UK), and anti-mouse IgG conjugated with Alexa-546 and anti-rabbit IgG conjugated Alexa-488 antibodies (A11003 and A11034, 1:1000; Thermo Fisher Scientific, Waltham, MA).

### HD model cells and mice

Neuro2a cell lines were stably transfected with truncated N-terminal huntingtin (tNhtt), which expresses a cDNA encoding huntingtin (htt) exon 1 containing 16 or 150 CAG repeats and was fused with enhanced green fluorescent protein (EGFP) with or without nuclear translocation signals (NLS). We generated four cell types including: tNhtt-16Q-EGFP (HD16Q cells), tNhtt-150Q-EGFP (HD150Q cells), tNhtt-16Q-EGFP-NLS (HD16Q-NLS cells), and tNhtt-150Q-EGFP-NLS (HD150Q-NLS cells). These cells were induced with 1 μM Ponasterone A and cultured as previously described [7]. Brain slices from two heterozygous htt exon 1 transgenic male mice of the R6/2 (145 CAG repeats) strain [Jackson code, B6CBA-TgN (HD exon1) 62] and two age-matched controls at 12 weeks of age were used for immunohistochemistry. The HD model cells and mouse brain samples were a kind gift from Dr. Nobuyuki Nukina (Doshisha University, Japan). All animal experiments conformed to the Guide for the Care and Use of Laboratory Animals.

### Human specimens

Human brain specimens were obtained during postmortem examination of patients with polyQ diseases (SCA1, SCA2, SCA3, and dentatorubral–pallidoluysian atrophy [DRPLA]), ALS, PD, and MSA (Table 1). Full consent from the family was obtained at the time of autopsy, and the Hospital Human Subjects Ethics Committee approved the study. Diagnoses were based on clinical and pathological findings. Human specimens were fixed in 10% formalin for 1 to 3 weeks, embedded in paraffin, and sectioned at a thickness of 5 μm.

**Table 1:** Clinical characteristics of postmortem cases

Disease	Case number	Age at death (years)	Sex (M/F)	Disease duration (months)	SGTA-reactive inclusions
SCA1	1	55	F	144	Positive
SCA1	2	71	F	492	Positive
SCA2	3	57	M	120	Positive
SCA2	4	48	F	252	Positive
SCA3	5	40	F	156	Positive
SCA3	6	51	F	240	Negative
SCA3	7	36	M	168	Positive
DRPLA	8	46	F	131	Positive
PD	9	82	F	155	Negative
PD	10	94	F	324	Negative
PD	11	82	F	66	Negative
PD	12	58	F	228	Negative
MSA	13	48	M	85	Positive
MSA	14	75	M	84	Positive
MSA	15	65	F	40	Positive
MSA	16	79	F	48	Positive
ALS	17	69	F	7	Negative
ALS	18	64	M	36	Negative
ALS	19	87	M	29	Negative
ALS	20	61	M	9	Negative
ALS	21	75	F	7	Negative
ALS	22	72	M	20	Negative

*M* male, *F* female, *SCA* spinocerebellar ataxia, *DRPLA* dentatorubral–pallidoluysian atrophy, *PD* Parkinson’s disease, *MSA* multiple system atrophy, *ALS* amyotrophic lateral sclerosis

### Plasmid construction

The cDNA for human SGTA was obtained from Mammalian Gene Collection cDNA clones (Invitrogen, Carlsbad, CA) and subcloned into the TOPO-pcDNA3.1/V5-His mammalian expression vector (Invitrogen). Using this SGTA vector as a template, SGTA deletion mutants were created by PCR and subcloned into the same vector. All constructs were verified by sequencing. Hdj1 plasmid was a kind gift from Dr. Nobuyuki Nukina (Doshisha University, Japan).

### Immunocytochemistry and immunohistochemistry

Immunostaining was performed as reported previously. [6] In brief, HD150Q cells or HD150Q-NLS cells were differentiated with 5 mM N^6^,2′-O-dibutyryladenosine-3′:5′-cyclic monophosphate sodium salt (dbcAMP, Nacalai, Kyoto, Japan) and induced to express tNhtt-polyQ with 1 μM Ponasterone A. After 2 days, immunocytochemistry was performed. The frozen mouse brain sections and paraffin-embedded human specimens were also subjected to immunohistochemistry as described previously [13]. Briefly, frozen mouse brain sections were washed twice with phosphate-buffered saline (PBS), fixed for 30 min with 100% methanol, washed three times PBS, blocked for 1 h with TBST containing 2% non-fat dried milk, and incubated overnight at 4 °C with primary antibodies. Then, the sections were washed three times with TBST and incubated with secondary antibody for 2 h, and detected using the Vectastain ABC kit (Vector Laboratories, Burlingame, CA). Human brain sections were autoclaved at 121 °C for 10 min, incubated in 100% formic acid for 5 min, and incubated with 1% hydrogen peroxidase for 15 min. The sections were then blocked for 1 h with TBST containing 7% goat serum, and then immunostained with primary antibodies followed by visualization using the Vectastain ABC kit. We confirmed the existence of polyQ inclusions with anti-expanded polyQ (1C2) antibody in polyQ disease cases (SCA1, SCA2, SCA3 and DRPLA), α-synuclein aggregates with anti-phosphorylated α-synuclein antibody in PD and MSA cases, and neuronal inclusions with anti-phosphorylated TDP-43 antibody in ALS cases. Then, SGTA immunoreactivity was investigated in relation to the localization of these pathological hallmarks in each case using double immunofluorescence staining visualized by a confocal laser scanning microscope (FV1000D, Olympus, Tokyo, Japan).

### Preparation of soluble and insoluble fractions from cell models

After HD model cells were differentiated and induced for 24 h, they were washed twice in ice cold PBS and harvested in lysis buffer (50 mM Tris–HCl [pH 7.5], 150 mM NaCl, 1% Triton X-100, 0.1% SDS, 1 mM EDTA, 0.5% sodium deoxycholate, and complete protease inhibitor cocktail [Roche, Basel, Switzerland]). Cell lysates were centrifuged at 10,000×*g* for 30 min and the resulting pellets were resuspended in lysis buffer. To prevent contamination of the soluble components by pellets, this process was repeated three times. The first supernatants and the final pellets were regarded as the soluble fractions and the insoluble fractions, respectively. The final pellets were then dissolved in urea buffer (30 mM Tris pH 8.5, 7 M urea and 2 M thiourea, 4% 3-[(3-Cholamidoproryl) dimenthylammonio]-1-propanesulfonate (CHAPS, 07957-51, Nacalai)). Each fraction was subjected to western blot analysis.

### Deletion assay

HD 150Q cells were transfected with the SGTA construct containing each deletion mutant using Lipofectamine LTX (Thermo Fisher Scientific) according to the manufacturer’s protocol. Twenty-four hours after transfection, these cells were differentiated and expression of tNhtt-150Q-GFP was induced. After another 24 h, immunocytochemistry was performed using anti-V5 antibody. Immunostaining was performed as reported previously [7].

### Filter trap assay

A filter trap assay was performed using a dot blot apparatus (SCIE-PLAS, Cambridge, UK) according to the method of Wanker et al. [14]. HD150Q cells were transfected with an expression vector construct for SGTA, LacZ or Hdj1. Twenty-four hours after transfection, cells were harvested and each cell lysate was filtered with a 0.2-μm-pore cellulose acetate membrane. After washing with PBS twice, membranes were analyzed by immunoblotting.

### Statistical analysis

For deletion assay and the filter trap assay, numerical comparison was performed with the Student’s t-test. Differences were considered significant at *P* < 0.05.

## Results

### SGTA colocalized with polyQ aggregates in HD model cells during aggregate formation

**Fig. 1:**
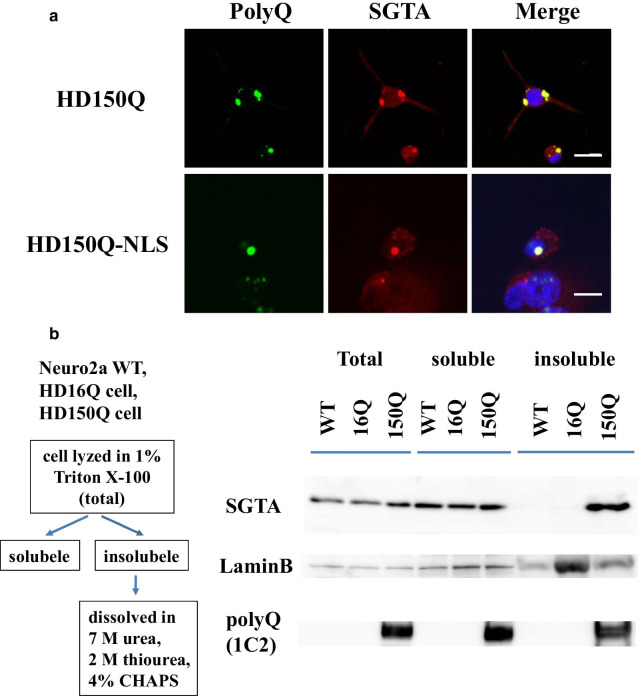
SGTA colocalized with polyQ aggregates in HD model cells. **a** Immunocytochemistry of Huntington disease (HD) model cells, HD150Q cells or HD 150QNLS cells. PolyQ aggregate formation was induced in these cells. Endogenous small glutamine-rich tetratricopeptide repeat (TPR)-containing protein alpha (SGTA) was labeled with anti-SGTA antibody (secondary antibody: Alexa Fluor 546). The nuclei were stained with 4′,6-diamidino-2-phenylindole (DAPI). Scale bar = 20 μm. **b** Cell lysates of Neuro2a wild type (WT), HD16Q, and HD150Q cells were fractionated into soluble and insoluble fractions. Immunoblotting with anti-SGTA, anti-LaminB and anti-1C2 antibodies was performed

First, we confirmed whether SGTA colocalizes with intracellular polyQ aggregates in HD model cells (HD150Q cells and HD150Q-NLS cells). Expanded polyQ-containing protein tNhtt-150Q or tNhtt-150Q-NLS formed aggregates in the cytoplasm or nucleus of HD model cells, respectively. In these cells, endogenous SGTA colocalized with cytoplasmic aggregates or intranuclear aggregates formed by the expanded polyQ-containing protein (Fig. 1a). In HD16Q and HD16Q-NLS cells, which were transfected with tNhtt-16Q or tNhtt-16Q-NLS, respectively, SGTA was diffusely distributed throughout the cytoplasm (Additional file 1, Fig. S1).

Next, we assessed SGTA biochemical properties in wild type Neuro2a, HD16Q, and HD150Q cells. In wild type and HD16Q cell lysates, SGTA was detected in the total and soluble fraction but not in the insoluble fraction, whereas SGTA was detected in both the soluble and insoluble fractions of HD150Q cells (Fig. 1b). These results suggest that SGTA shifted from soluble to insoluble upon aggregate formation.

### SGTA associated with aggregates in HD model mice

**Fig. 2:**
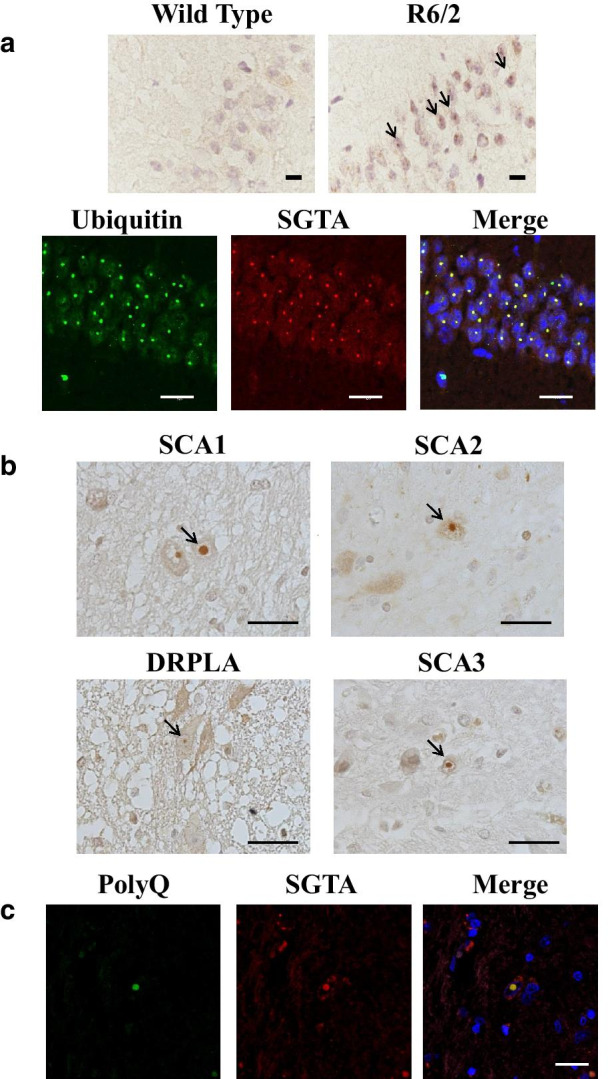
SGTA colocalized with neuronal intranuclear inclusions (NIIs) in postmortem brain sections of polyQ diseases. **a** Upper panels show immunohistochemistry of frozen brain sections of wild type (left) and R6/2 (right) mice using anti-SGTA antibody. Scale bars = 10 μm. Lower panels show double immunostaining images of the CA1 region in the hippocampus of the R6/2 mouse (left: ubiquitin, middle: SGTA, right: merged). Scale bars = 20 μm. **b** Immunohistochemistry with anti-SGTA antibody in the pontine nucleus of postmortem tissue from patients with polyQ diseases (spinocerebellar ataxias [SCA1, SCA2, SCA3] and dentatorubral–pallidoluysian atrophy [DRPLA]). NIIs indicated by arrow. Scale bar = 20 μm. **c** Double immunohistochemical labeling with the anti-polyQ antibody (green) and anti-SGTA antibody (red) in the pontine nucleus of postmortem tissue from a patient with SCA1. The nuclei were stained with DAPI. Scale bar = 20 μm

Next, we investigated whether SGTA colocalizes with polyQ aggregates in R6/2 mice using immunohistochemistry. SGTA associated with neuronal intranuclear inclusions (NIIs) in the CA1 region of mouse brains (Fig. 2a). This colocalization was confirmed by double immunofluorescence staining with antibodies against SGTA and ubiquitin. In control mouse brain, SGTA was diffusely distributed throughout the cytoplasm of neurons.

### SGTA colocalized with neuronal intranuclear inclusions in patients with polyQ diseases

Next, we examined whether SGTA associates with NIIs in postmortem brains of patients with polyQ diseases. We performed immunohistochemistry on postmortem brain sections from eight patients with SCA1 (n = 2), SCA2 (n = 2), SCA3 (n = 3), and DRPLA (n = 1). Immunohistochemistry showed that SGTA localized to NIIs in the pontine nucleus of brains from all of the polyQ disease patients except one patient with SCA3 (case 6 in Table 1; Fig. 2b). Furthermore, double immunofluorescence staining using anti-SGTA antibody and anti-expanded polyQ antibody (1C2) was performed. In the brain of a patient with SCA1, SGTA colocalized with NIIs in the pontine nucleus (Fig. 2c), indicating an association of SGTA with polyQ aggregates. SGTA modulates intracellular protein quality control in cooperation with the BCL2-associated athanogene 6 (BAG6) complex, therefore we also examined whether BAG6 associates with NIIs in postmortem brains of patients with polyQ diseases. BAG6 was not detected in aggregates in HD model mouse brains and postmortem polyQ disease brains (Additional file 1, Fig. S2).

### SGTA colocalized with glial cytoplasmic inclusions in patients with MSA

**Fig. 3:**
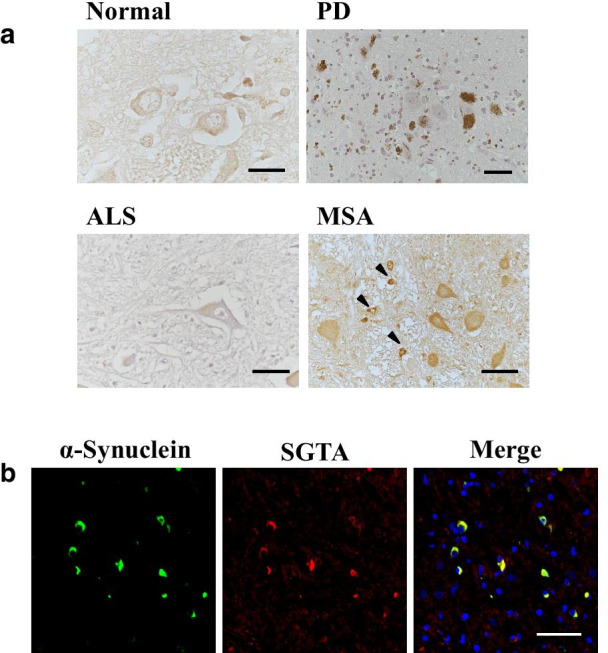
SGTA interacted with glial cytoplasmic inclusions (GCIs) in multiple system atrophy (MSA). **a** Immunohistochemistry of postmortem tissue with anti-SGTA antibody in the substantia nigra of PD patients, in the medulla of MSA patients, in the spinal cord of ALS patients, and in the pontine nucleus of control patients. Control patients: scale bar = 20 μm, others: scale bar = 50 μm. Note that intense staining observed in the substantia nigra of PD patients represents the melanin pigments. Arrow heads indicate SGTA staining of GCIs in oligodendrocytes. **b** Double fluorescence immunohistochemistry labeling with anti-phosphorylated α-synuclein antibody (green) and anti-SGTA antibody (red) in the medulla of a patient with MSA. Scale bar = 50 μm

We also performed immunohistochemistry on tissue from four PD patients and four MSA patients, and on spinal cord from six ALS patients. In the MSA brains, SGTA was diffusely distributed in the cytoplasm of neurons. Strong SGTA staining was also detected in the cytoplasm of glial cells, showing similar distribution to glial cytoplasmic inclusions (GCIs), a pathological hallmark of MSA (Fig. 3a). Double immunofluorescence staining using anti-SGTA antibody and anti-phosphorylated α-synuclein antibody revealed that SGTA colocalized with phosphorylated α-synuclein in GCIs (Fig. 3b). In brains of PD patients, SGTA was diffusely detected in the cytoplasm of neurons (Fig. 3a). Double immunofluorescence staining with anti-phosphorylated α-synuclein and anti-SGTA antibodies showed that SGTA did not colocalize with phosphorylated α-synuclein-reactive portions of Lewy bodies. However, in the cases with some mature Lewy bodies with halo structure, SGTA antibody weakly reacted with the core where no phosphorylated α-synuclein reactivity was detected (Additional file 1, Fig. S3). In spinal cords of ALS patients, SGTA was diffusely distributed in the cytoplasm of neurons (Fig. 3a), and double immunofluorescence staining with anti-phosphorylated TDP-43 antibody revealed that neuronal cytoplasmic inclusions of ALS were not stained by SGTA antibody (Additional file 1, Fig. S4).

### SGTA interacts with polyQ aggregates through its C-terminal domain

**Fig. 4:**
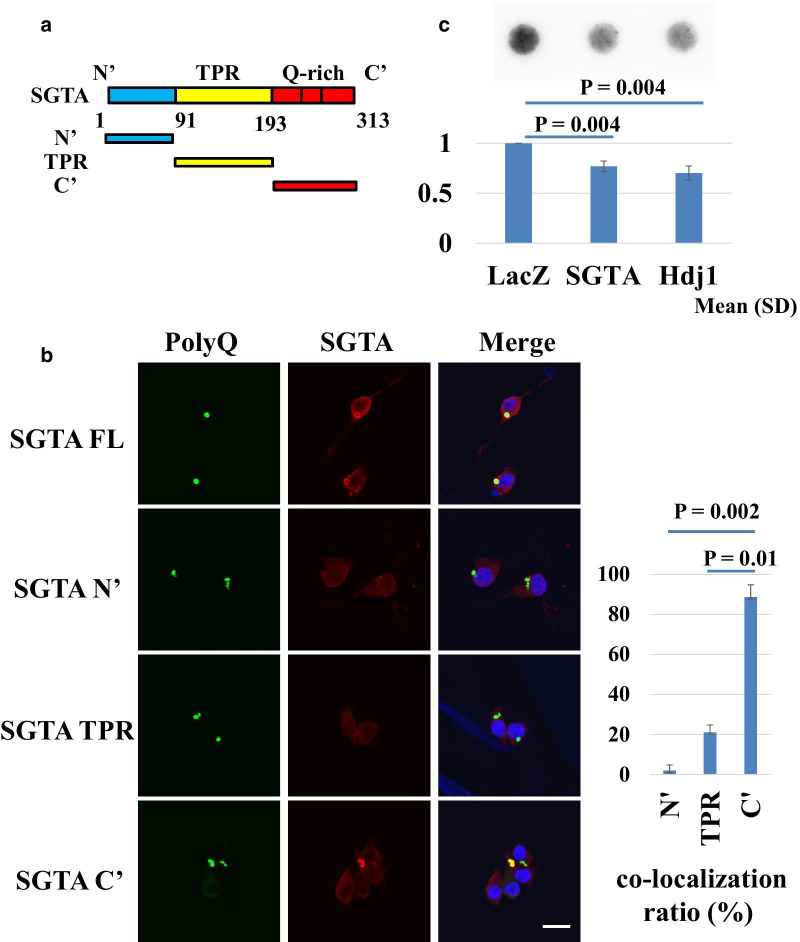
C-terminal domain of SGTA interacted with aggregates in HD model cells. **a** Schema of the full-length SGTA and deletion mutant constructs. **b** Immunocytochemistry of HD150Q cells transfected with expression vector of full-length SGTA or deletion mutants. Each expression protein was tagged with V5 protein and labeled with anti-V5 antibody. Scale bar = 20 μm. Ratio of SGTA colocalization with polyQ aggregates is also shown. **c** Filter trap assay for comparing aggregate formation in SGTA-, LacZ- (negative control), and Hdj1- (positive control) overexpressing cells. HD150 cells were transfected with each vector and cell lysates were subjected to filter trap assay. Experiments were performed three times and statistical significance was assessed by Student’s T-test

Next, to identify the SGTA domain that interacts with aggregates, HD150Q cells were transfected with expression plasmids encoding V5-tagged full-length SGTA and three types of SGTA deletion mutants (Fig. 4a). Immunocytochemistry showed that full-length SGTA and the SGTA C-terminal domain colocalized with polyQ aggregates (Fig. 4b). Deletion mutants of SGTA protein containing the N-terminal or TPR domains were diffusely expressed in cytoplasm without aggregate formation. We counted the number of SGTA-positive aggregates among the polyQ aggregates in cells transfected with deletion mutants of SGTA. C-terminal domain of SGTA-transfected cells showed the most abundant SGTA-positive polyQ aggregates, suggesting that the C-terminal domain of SGTA interacts with polyQ aggregates (Fig. 4b). Next, HD16Q and HD150Q cell lysates were immunoprecipitated with anti-GFP antibody and subjected to immunoblot analysis with anti-SGTA antibody. We did not observe interaction of tNhtt-polyQ and SGTA (Additional file 1, Fig. S5).

### SGTA overexpression reduced polyQ aggregates in HD model cells

Finally, we assessed the effect of overexpression of SGTA on intracellular aggregate formation. HD150Q cells were transfected with SGTA, LacZ, or Hdj1 expression plasmid vectors. The levels of aggregates in SGTA-transfected cells were significantly lower than those in LacZ-transfected cells (Fig. 4c). Hdj1, a heat shock protein family member, was used as a positive control for aggregate formation suppression [4] and also demonstrated reduced aggregate formation in HD model cells. The densitometric analysis showed levels of aggregates in SGTA- and Hdj1-overexpressing cells were 77.0 ± 5.3%, *P* = 0.004 and 70.3 ± 7.0%, *P* = 0.004 (mean ± SEM; *n* = 6 in each case) lower, respectively, than levels in LacZ-transfected cells. We also counted and compared the number of polyQ aggregate-positive cells among the cells transfected with each plasmid vector (LacZ, SGTA or Hdj1). In SGTA-transfected cells, the percentage of aggregate-positive cells did not significantly change compared with the percentage in LacZ-transfected cells (Additional file 1, Fig. S6A). We further performed flow cytometric analysis using HD150Q cells treated in the same manner as above and the results disclosed two GFP fluorescent peaks (weak fluorescence and strong fluorescence; Additional file 1, Fig. S6B and S6C). SGTA and Hdj1 both reduced the weak fluorescent peaks (red arrows, Additional file 1, Fig. S6B and S6C), whereas they did not affect the strong fluorescent peaks.

## Discussion

In this study, we showed that SGTA is a component of polyQ aggregates. Immunostaining demonstrated that SGTA colocalized with polyQ aggregates in HD model cells and HD model mice. SGTA also associated with NIIs in patients with polyQ diseases including SCA1, SCA2, SCA3, and DRPLA. Additionally, SGTA interacted with GCIs in postmortem tissue of patients with MSA. SGTA overexpression enhanced solubility of polyQ aggregates in HD model cells.

SGTA, originally identified as an interacting partner for nonstructural protein NS1 of parvovirus H-1 [15], has been shown to interact with the C-terminal of Hsc70 by TPR domains [16], and also with the synaptic vesicle cysteine string protein (CSP) to form a stable trimeric complex that functions as a chaperone machine [17]. Additional roles of SGTA have been reported, such as in the regulation of steroid hormone receptors [18, 19] and modulation of the protein quality control system by working with the BAG6 complex [9, 10]. Depletion of SGTA causes accumulation of BAG6-dependent ERAD substrates, indicating that SGTA assists BAG6 functions to prevent the formation of nondegradable protein aggregates by ERAD [9]. In contrast, SGTA has also been reported to antagonize the actions of BAG6 and delay the proteasomal degradation of mislocalized membrane proteins (MLPs) by promoting the deubiquitination of MLPs [20–22]. Possible involvement of SGTA in neurodegeneration has been suggested based on the observation that the SGTA ortholog binds with intracellular Aβ in a *C. elegans* model expressing a chimeric signal peptide/human Aβ1-42 [23]. In addition, CSP, the binding partner of SGTA, has been shown to bind the huntingtin fragment with an expanded polyQ region [24]. Considering these findings in disease models, and the perturbation of cellular protein homeostasis and accumulation of aberrant proteins as important factors in pathophysiology of neurodegenerative diseases [25, 26], SGTA may be involved in the pathogenesis of neurodegenerative diseases. To the best of our knowledge, this study provides the first evidence that SGTA is associated with intracellular aggregates in postmortem brains of individuals with polyQ diseases (SCA1, SCA2, SCA3 and DRPLA) and MSA.

SGTA has been reported to associate with the hydrophobic domain of membrane proteins through its C-terminal domain [27, 28], and to cooperate with their binding partners to maintain cytosolic protein quality control [9]. In this study, immunohistochemical analysis indicated that SGTA interacts with polyQ aggregates through the C-terminal domain. This finding is consistent with previous studies because expanded polyQ tracts also have hydrophobic properties [29]. Immunoprecipitation of HD16Q and HD150Q cell lysates with anti-GFP antibody failed to reveal interaction with SGTA (Additional file 1, Fig. S5), presumably because anti-GFP antibody only precipitates soluble monomers or oligomers of tNhtt-polyQ. Thus, our results suggest that SGTA interacts with tNhtt-polyQ after the formation of insoluble aggregates.

We also showed that SGTA overexpression enhanced solubility of intracellular aggregates in HD model cells, suggesting that SGTA alters the properties of aberrant polyQ proteins (Fig. 4c). Flow cytometric analysis indicated that SGTA and Hdj1 affected the size distributions of polyQ aggregates in a similar fashion (Additional file 1, Fig. S6B and S6C). Both proteins reduced only small and presumably insoluble polyQ aggregates (i.e. weak fluorescent signals), but not large and matured poly Q aggregates (i.e. strong fluorescent signals), which may account for the discrepant results between filter trap assay (Fig. 4c) and fluorescent microscopic analysis, mainly reflecting the status of large aggregates (Additional file 1, Fig. S6A).

BAG6 was undetected in aggregates of HD model mouse brains and postmortem polyQ disease brains, suggesting that SGTA may affect polyQ aggregates using alternative pathways distinct from BAG6-mediated pathways.

SGTA also colocalized with GCIs in oligodendrocytes of MSA brains (Fig. 3), but not with phosphorylated α-synuclein-positive portions of Lewy bodies in neurons of PD brains (Additional file 1, Fig. S3). Although both GCIs and Lewy bodies are mainly composed of α-synuclein, these two strains of aggregates have different characteristics, such as conformation and cell toxicity [30]. It is also possible that the accumulation process and protein processing pathway of α-synuclein might be different between neurons and oligodendrocytes. The findings suggest that SGTA may be involved in pathophysiological mechanisms for various polyQ diseases and MSA, but not necessarily for all neurodegenerative diseases including PD and ALS. Further study is required to clarify the role of SGTA in neurodegenerative processes.

In summary, we showed that SGTA is an aggregate-interacting protein in polyQ diseases and MSA. SGTA was found in polyQ-positive NIIs and α-synuclein-positive GCIs. These findings, together with the known role of SGTA in intracellular protein homeostasis, suggest that SGTA may play an essential role in the aggregate accumulation process in these diseases. However, further study is needed to elucidate the role of SGTA in pathophysiological mechanisms of neurodegenerative diseases.

## Supplementary Information

**Additional file 1: Figure S1**. Distribution of SGTA in HD16Q and HD16Q-NLS cells. **Figure S2.** BAG6 in brains of an HD model mouse and human polyglutamine diseases. **Figure S3.** SGTA in PD brain. **Figure S4.** SGTA in motor neurons in ALS spinal cords. **Figure S5.** Immunoprecipitation of tNhtt-polyQ proteins.**Figure S6.** HD150Q cells transfected with LacZ, SGTA, or Hdj1.

**Additional file 2:** Supplementary materials and methods, supplementary figure legends and supplementary reference.

## Data Availability

The datasets used during the current study are available from the corresponding author on reasonable request.

## Abbreviations

ALS: Amyotrophic lateral sclerosis
BAG6: BCL2-associated athanogene 6
DAPI: 4′,6-Diamidino-2-phenylindole
CSP: Cysteine string protein
dbc-AMP: N^6^,2′-O-dibutyryladenosine-3′:5′-cyclic monophosphate sodium salt
DRPLA: Dentatorubral–pallidoluysian atrophy
EGFP: Enhanced green fluorescent protein
ERAD: Endoplasmic reticulum-associated degradation
GCIs: Glial cytoplasmic inclusions
HD: Huntington disease
MLP: Mislocalized membrane proteins
MSA: Multiple system atrophy
NIIs: Neuronal intranuclear inclusions
NLS: Nuclear translocation signals
PBS: Phosphate-buffered saline
PD: Parkinson’s disease
PolyQ: Polyglutamine
SCA: Spinocerebellar ataxia
SGTA: Small glutamine-rich tetratricopeptide repeat-containing protein alpha
tNhtt: Truncated N-terminal huntingtin
TPR: Tetratricopeptide repeat
