# Supplementary material for: SGTA associates with intracellular aggregates in neurodegenerative diseases
Source: Mol Brain. 2021 Mar 23;14:59. doi: 10.1186/s13041-021-00770-1 (PMC7986274; doi:10.1186/s13041-021-00770-1)
Supplement: Supplementary file 1 — Additional file 1: Figure S1. Distribution of SGTA in HD16Q and HD16Q-NLS cells. Figure S2. BAG6 in brains of an HD model mouse and human polyglutamine diseases. Figure S3. SGTA in PD brain. Figure S4. SGTA in motor neurons in ALS spinal cords. Figure S5. Immunoprecipitation of tNhtt-polyQ proteins.Figure S6. HD150Q cells transfected with LacZ, SGTA, or Hdj1. [file 13041_2021_770_MOESM1_ESM.pdf]

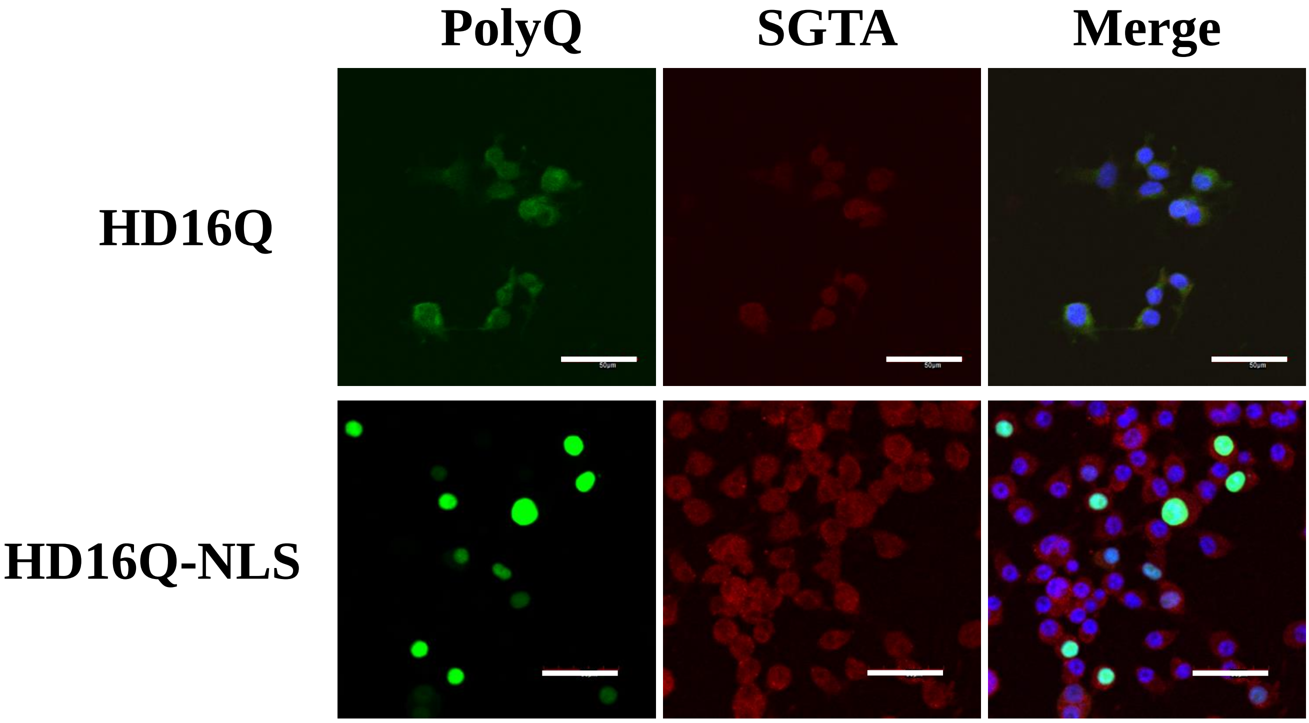

Supplementary Figure. 1

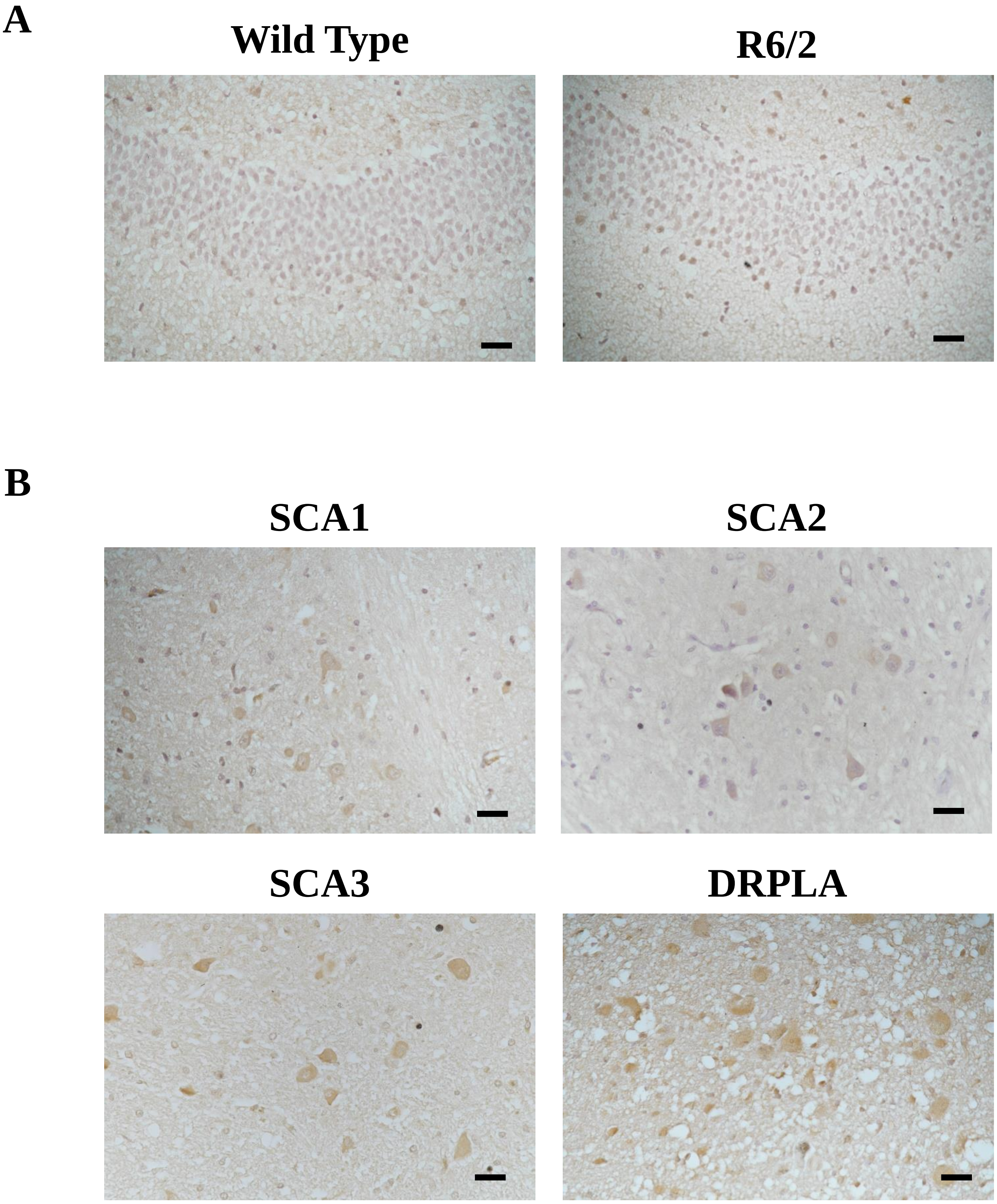

Supplementary Figure. 2

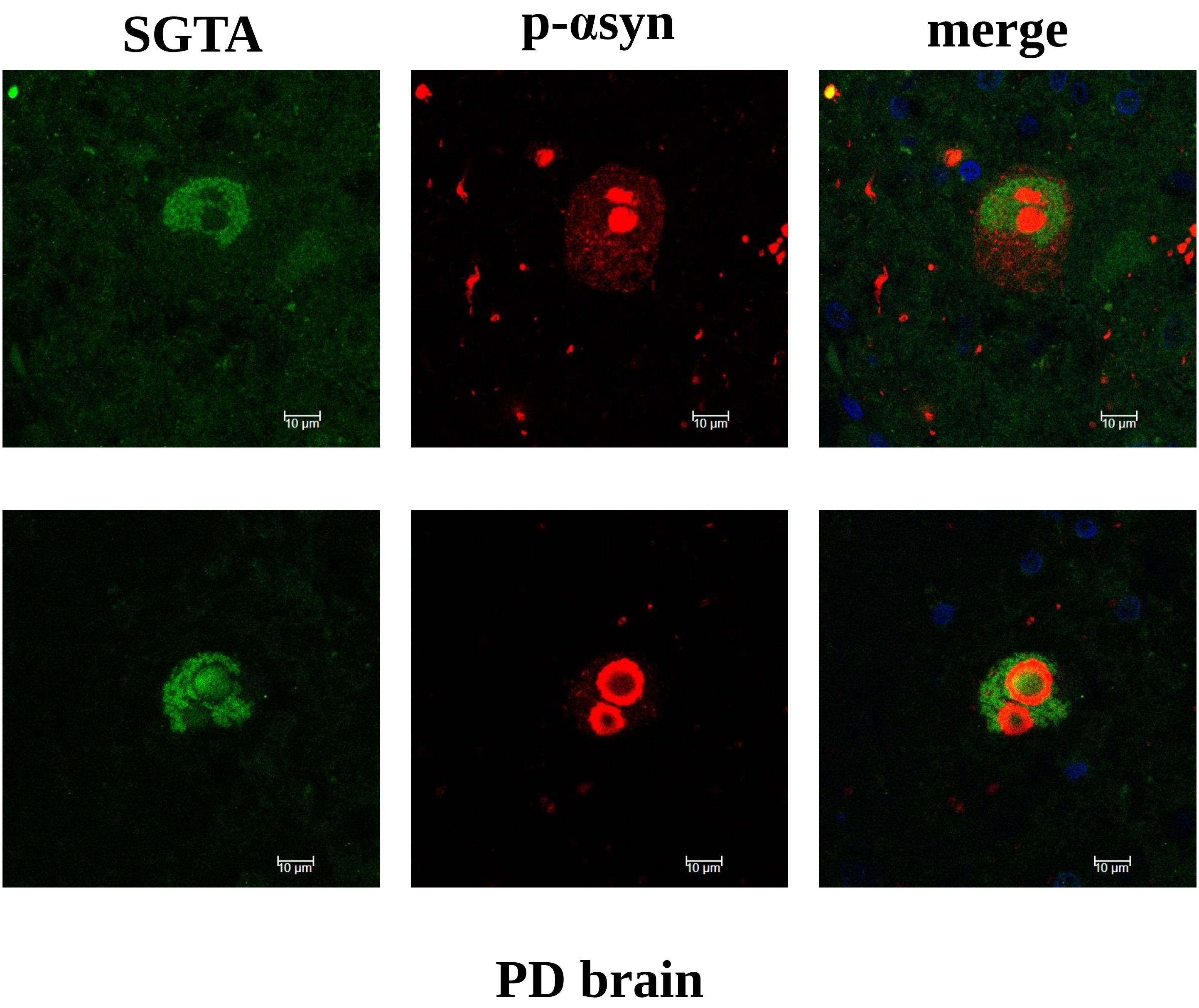

Supplementary Figure. 3

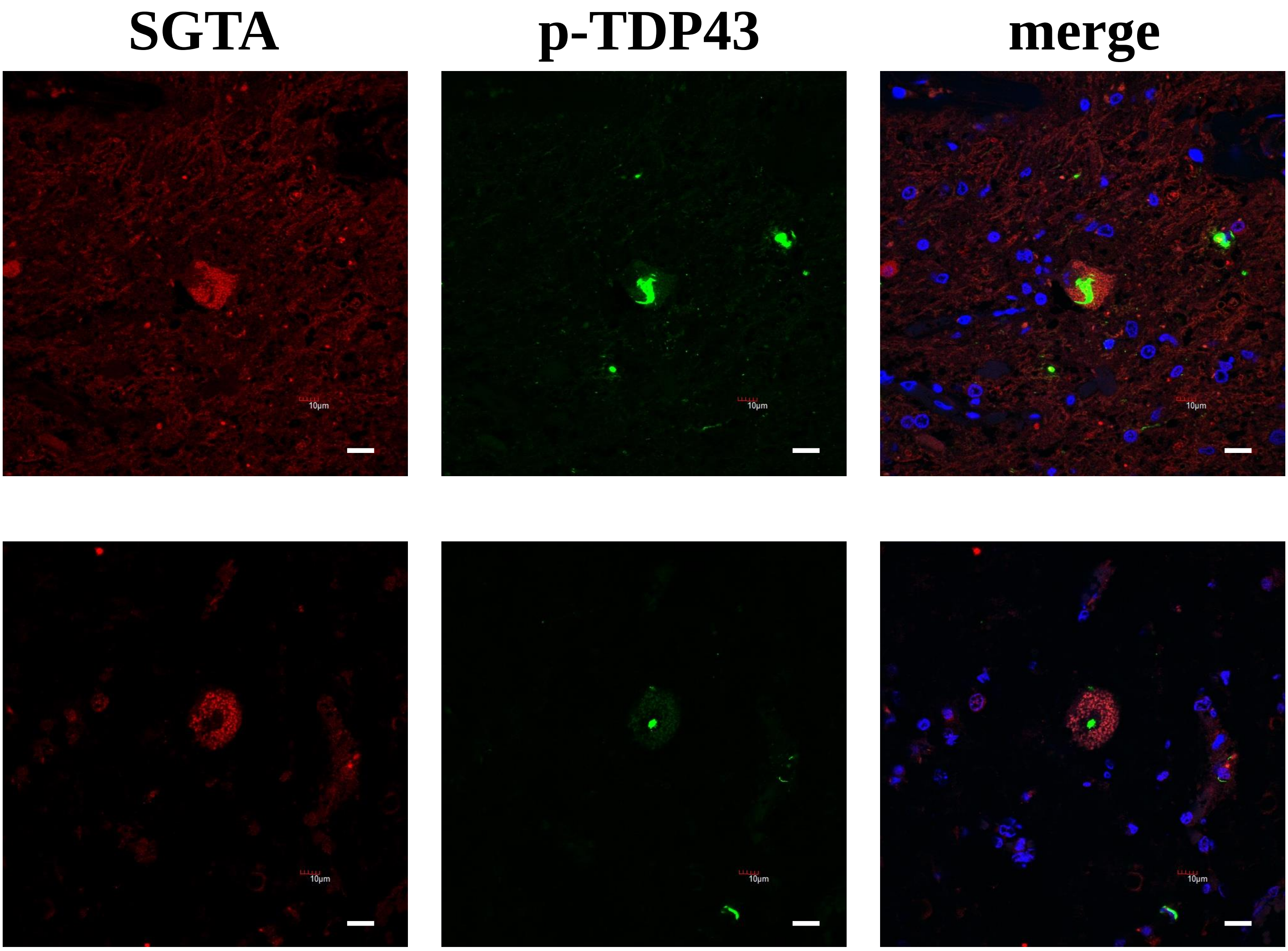

ALS spinal cord

Supplementary Figure. 4

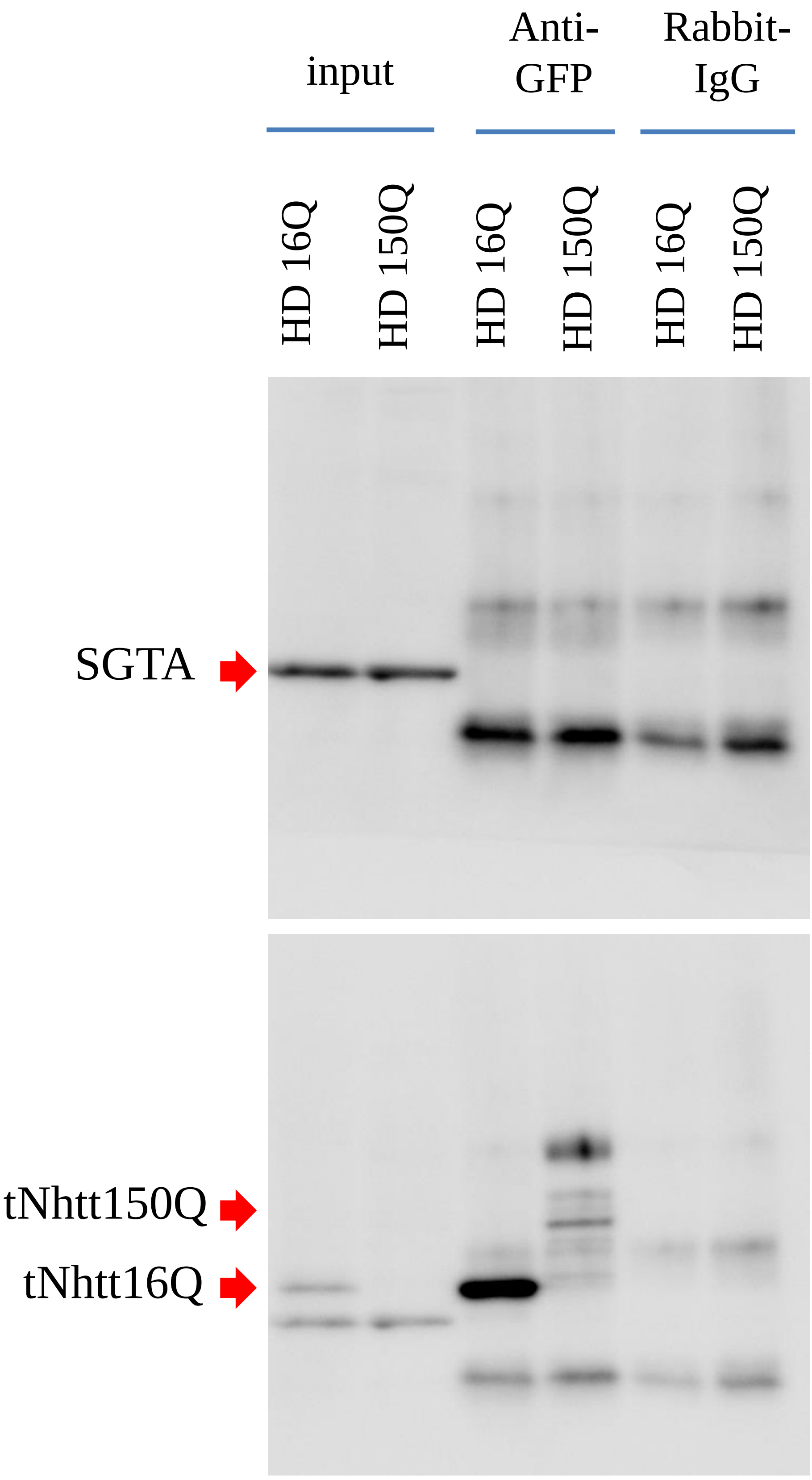

Supplementary Figure. 5

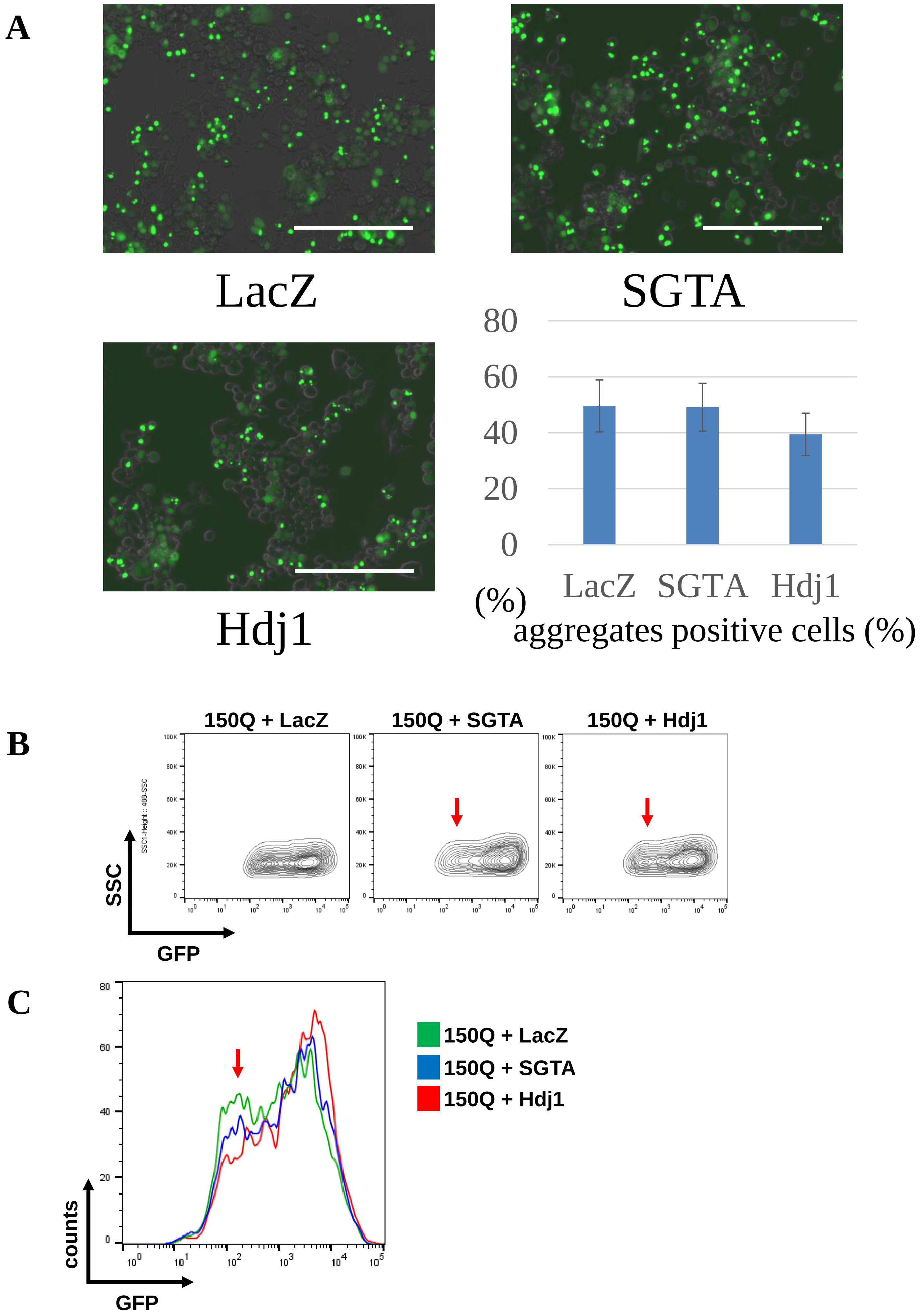

Supplementary Figure. 6
