# Supplementary material for: SGTA associates with intracellular aggregates in neurodegenerative diseases
Source: Mol Brain. 2021 Mar 23;14:59. doi: 10.1186/s13041-021-00770-1 (PMC7986274; doi:10.1186/s13041-021-00770-1)
Supplement: Supplementary file 2 — Additional file 2: Supplementary materials and methods, supplementary figure legends and supplementary reference. [file 13041_2021_770_MOESM2_ESM.docx]

***Supplementary information***

**Title: SGTA associates with intracellular aggregates in neurodegenerative diseases.**

**Authors:**

Shun Kubota, M.D.^1^ e-mail: t146032f@yokohama-cu.ac.jp

Hiroshi Doi, M.D., Ph.D.^1^ e-mail: hdoi@yokohama-cu.ac.jp

Shigeru Koyano, M.D., Ph.D.^1, 2^ e-mail: korokkekoyano@gmail.com

Kenichi Tanaka, M.D., Ph.D.^1^ e-mail: [ktanaka-2@hotmail.co.jp](mailto:ktanaka-2@hotmail.co.jp)

Hiroyasu Komiya, M.D. ^1^ e-mail: verdydoniz@yahoo.co.jp

Atsuko Katsumoto, M.D., Ph.D.^1^ e-mail: chiwawan-atsuko@hotmail.co.jp

Shingo Ikeda, M.D.^1^ e-mail: ikedashingo29@gmail.com

Shunta Hashiguchi, M.D., Ph.D.^1^ e-mail: shashig@yokohama-cu.ac.jp

Haruko Nakamura, M.D., Ph.D.^1^ e-mail: haruko0224@msn.com

Ryoko Fukai, M.D., Ph.D.^1^ e-mail: ryokofukai@gmail.com

Keita Takahashi, M.D., Ph.D.^1^ e-mail: interferon1234@gmail.com

Misako Kunii, M.D., Ph.D. ^1^ e-mail: seaweed335@msn.com

Mikiko Tada, M.D., Ph.D.^1^ e-mail: mikikosug@gmail.com

Hideyuki Takeuchi, M.D., Ph.D.^1^ e-mail: htake@yokohama-cu.ac.jp

Fumiaki Tanaka, M.D., Ph.D. ^1^ e-mail: ftanaka@yokohama-cu.ac.jp

^1^Department of Neurology and Stroke Medicine, Yokohama City University Graduate School of Medicine, 3-9 Fukuura, Kanazawa-ku, Yokohama 236-0004, Japan

^2^Department of Neurology, Yokohama Minami Kyosai Hospital, 1-21-1 Mutuurahigashi, Kanazawa-ku, Yokohama, 236-0037, Japan

**Corresponding authors:** Hiroshi Doi and Fumiaki Tanaka

**Supplementary materials and methods**

**Immunocytochemistry and immunohistochemistry**

Immunocytochemistry and immunohistochemistry were performed in the same manner as described in the main text.

**Immunoprecipitation assay**

Immunoprecipitation was performed as reported previously^1^. Briefly, HD16Q cells or HD150Q cells were differentiated with 5 mM dbcAMP and induced to express tNhtt-polyQ with 1 μM Ponasterone A. After 24 h, these cells were harvested and suspended in lysis buffer (50 mM Tris-HCl [pH 7.5], 150 mM NaCl, 1% Triton X-100, 0.1% SDS, 1 mM EDTA, 0.5% sodium deoxycholate and complete protease inhibitor cocktail) on ice for 30 min. Cell lysates were homogenized by 30 strokes with a Dounce glass homogenizer and centrifuged at 10,000 g for 30 min. The supernatants were used for immunoprecipitation. Cell lysates containing 500 μg protein in 500 μl volume were incubated with 2 μl (5 μg) of anti-GFP antibody or 2 μl (5 μg) of normal rabbit IgG for 6 h at 4 °C with rotation and 100 μl of magnetic protein G beads (Thermo Fisher Scientific, Waltham, MA) were added, followed by further overnight incubation at 4 °C. The beads were pulled down with a magnetic rack and washed six times with lysis buffer. After that, bound proteins were eluted with SDS sample buffer (62.5 mM Tris-HCl [pH 6.8], 2% SDS, 10% glycerol, 5% 2-mercaptoethanol, 0.02% bromophenol blue) from the beads, boiled for 5 min at 98 °C and subjected to immunoblotting using anti-SGTA antibody.

**Aggregate counting**

HD150Q cells were transfected with each expression plasmid vector (LacZ, SGTA or Hdj1) and PolyQ aggregate formation was induced with 1 μM Ponasterone A and differentiated with 5 mM dbc-AMP. After 48 h from transfection, aggregate-positive cells were counted using a fluorescence microscope (EVOS FL Cell Imaging System, Thermo Fischer Scientific) and the percentage of positivity was calculated and statistically analyzed by Student’s t-test.

**Flow cytometric analysis**

HD150Q cells were transfected with an expression vector construct for LacZ, SGTA or Hdj1. These cells were differentiated and expression of tNhtt-150Q-GFP was induced. Twenty-four hours after transfection, cells were harvested and GFP-positive cells (> 5,000 cells) were analyzed by a flow cytometer (MoFlo Astrios, Beckman Coulter, Fullerton, CA) and FlowJo software (Becton Dickinson, Franklin Lakes, NJ).

**Supplementary figure legends**

**Figure S1. Distribution of SGTA in HD16Q and HD16Q-NLS cells.** Expression of tNhtt-PolyQ proteins was induced in HD16Q and HD16Q-NLS cells. Endogenous SGTA was labeled with anti-SGTA antibody (secondary antibody: Alexa Fluor 546). The nuclei were stained with 4′,6-diamidino-2-phenylindole (DAPI). SGTA was distributed throughout the cytoplasm in HD16Q and HD16Q-NLS cells. Scale bar = 50 μm.

**Figure S2. BAG6 in brains of an HD model mouse and human polyglutamine diseases**

(A) Immunohistochemistry of frozen brain sections of wild type (left) and R6/2 (right) mice using anti-BAG6 antibody. Scale bars = 25 μm. (B) Immunohistochemistry with anti-BAG6 antibody in the pontine nucleus of postmortem tissue from patients with polyglutamine **(**polyQ) diseases (spinocerebellar ataxias [SCA1, SCA2, SCA3] and dentatorubral–pallidoluysian atrophy [DRPLA]). BAG6 was not detected in aggregates of an HD model mouse and postmortem polyQ disease brains. Scale bar = 25 μm.

**Figure S3. SGTA in PD brain**

Double fluorescence immunohistochemistry labeling with anti-phosphorylated α-synuclein antibody (red) and anti-SGTA antibody (green) in the substantia nigra of PD patients. SGTA did not show reactivity with phosphorylated α-synuclein-positive Lewy bodies. Scale bar = 10 μm.

**Figure S4. SGTA in motor neurons in ALS spinal cords**

Double fluorescence immunohistochemistry labeling with anti-phosphorylated TDP-43 antibody (green) and anti-SGTA antibody (red) in the spinal cord of an ALS patient. SGTA did not colocalize with phosphorylated TDP-43-reactive neuronal inclusions. Scale bar = 10 μm.

**Figure S5. Immunoprecipitation of tNhtt-polyQ proteins**

GFP-fused tNhtt-16Q and tNhtt-150Q proteins were immunoprecipitated by anti-GFP antibody from HD16Q and HD150Q cell lysates, respectively. Protein was detected by immunoblot with anti-SGTA antibody (upper panel) or anti-GFP antibody (lower panel).

**Figure S6. HD150Q cells transfected with LacZ, SGTA, or Hdj1**

(A) HD150Q cells were transfected with each vector (LacZ, SGTA or Hdj1) and GFP-fused polyQ aggregate formation was induced. The percentage of aggregate-positive cells was calculated and the results were subjected to statistical analysis. Scale bars = 50 μm. (B) Contour plots of flow cytometric data for the transfected cells. X-axes and Y-axes indicate GFP fluorescent signals and side scatter (SSC), respectively. HD150Q cells showed two GFP fluorescence peaks (weak fluorescence and strong fluorescence). SGTA and Hdj1 both reduced the weak fluorescent peaks (red arrows). (C) Histograms of B for the transfected cells. X-axis and Y-axis indicate GFP fluorescent signals and counts, respectively. SGTA and Hdj1 both reduced the weak fluorescent peaks (red arrow).

**Supplementary reference**

1. Jana NR, Tanaka M, Wang G, Nukina N. Polyglutamine length-dependent interaction of Hsp40 and Hsp70 family chaperones with truncated N-terminal huntingtin: their role in suppression of aggregation and cellular toxicity. Hum Mol Genet. 2000;9:2009–18.
